# Obtaining evidence base for the development of Feel4Diabetes intervention to prevent type 2 diabetes – a narrative literature review

**DOI:** 10.1186/s12902-019-0468-y

**Published:** 2020-03-12

**Authors:** Jemina Kivelä, Katja Wikström, Eeva Virtanen, Michael Georgoulis, Greet Cardon, Fernando Civeira, Violeta Iotova, Ernest Karuranga, Winne Ko, Stavros Liatis, Konstantinos Makrilakis, Yannis Manios, Rocío Mateo-Gallego, Anna Nanasi, Imre Rurik, Tsvetalina Tankova, Kaloyan Tsochev, Vicky Van Stappen, Jaana Lindström, on behalf of the Feel4Diabetes research group, Yannis Manios, Greet Cardon, Jaana Lindström, Peter Schwarz, Konstantinos Makrilakis, Lieven Annemans, Ignacio Garamendi, Meropi Kontogianni, Odysseas Androutsos, Christina Mavrogianni, Konstantina Tsoutsoulopoulou, Christina Katsarou, Eva Karaglani, Irini Qira, Efstathios Skoufas, Konstantina Maragkopoulou, Antigone Tsiafitsa, Irini Sotiropoulou, Michalis Tsolakos, Effie Argyri, Mary Nikolaou, Eleni-Anna Vampouli, Christina Filippou, Katerina Gatsiou, Efstratios Dimitriadis, Tiina Laatikainen, Katja Wikström, Jemina Kivelä, Päivi Valve, Esko Levälahti, Eeva Virtanen, Vicky Van Stappen, Nele Huys, Ruben Willems, Samyah Shadid, Ivonne Panchyrz, Maxi Holland, Patrick Timpel, Stavros Liatis, George Dafoulas, Christina-Paulina Lambrinou, Angeliki Giannopoulou, Lydia Tsirigoti, Evi Fappa, Costas Anastasiou, Konstantina Zachari, Lala Rabemananjara, Maria Stella de Sabata, Winne Ko, Luis Moreno, Fernando Civeira, Gloria Bueno, Pilar De Miguel-Etayo, Esther Mª. Gonzalez-Gil, Maria I. Mesana, Germán Vicente-Rodriguez, Gerardo Rodriguez, Lucia Baila-Rueda, Ana Cenarro, Estíbaliz Jarauta, Rocío Mateo-Gallego, Violeta Iotova, Tsvetalina Tankova, Natalia Usheva, Kaloyan Tsochev, Nevena Chakarova, Sonya Galcheva, Rumyana Dimova, Yana Bocheva, Zhaneta Radkova, Vanya Marinova, Yuliya Bazdarska, Tanya Stefanova, Imre Rurik, Timea Ungvari, Zoltán Jancsó, Anna Nánási, László Kolozsvári, Csilla Semánova, Remberto Martinez, Marcos Tong, Kaisla Joutsenniemi, Katrina Wendel-Mitoraj

**Affiliations:** 1 Department of Public Health Solutions, National Institute for Health and Welfare, PO BOX 27, 00300 Helsinki, Finland; 2 Department of Obstetrics and Gynecology, University of Helsinki, Helsinki University Hospital, Helsinki, Finland; 3 Institute of Public Health and Clinical Nutrition, University of Eastern Finland, Kuopio, Finland; 4 Department of Nutrition and Dietetics, Harokopio University, Athens, Greece; 5 Department of Movement and Sports Sciences, Ghent University, Ghent, Belgium; 6 GENUD (Growth, Exercise, Nutrition and Development) Research Group, University of Zaragoza, Zaragoza, Spain; 7 Department of Paediatrics, Medical University Varna, Varna, Bulgaria; 8 International Diabetes Federation, Brussels, Belgium; 9 National and Kapodistrian University of Athens, Athens, Greece; 10 Department of Family and Occupational Medicine, Faculty of Public Health, University of Debrecen, Debrecen, Hungary; 11 Department of Diabetology, Clinical Center of Endocrinology, Medical University Sofia, Sofia, Bulgaria

**Keywords:** Families, Lifestyle intervention, Prevention, Risk factors, Type 2 diabetes, Vulnerable

## Abstract

**Background:**

Feel4Diabetes was a school and community based intervention aiming to promote healthy lifestyle and tackle obesity for the prevention of type 2 diabetes among families in 6 European countries. We conducted this literature review in order to guide the development of evidence-based implementation of the Feel4Diabetes intervention. We focused on type 2 diabetes prevention strategies, including all the phases from risk identification to implementation and maintenance. Special focus was given to prevention among vulnerable groups and people under 45 years.

**Methods:**

Scientific and grey literature published between January 2000 and January 2015 was searched for relevant studies using electronic databases. To present the literature review findings in a systematic way, we used the Reach, Effectiveness, Adoption, Implementation, and Maintenance (RE-AIM) framework. A complementary literature search from February 2015 to December 2018 was also conducted.

**Results:**

The initial review included 27 studies with a follow-up ≥12 months and 9 studies with a follow-up ≥6 months and with a participant mean age < 45 years. We found out that interventions should be targeted at people at risk to improve recruiting and intervention effectiveness. Screening questionnaires (primarily Finnish Diabetes Risk Score FINDRISC) and blood glucose measurement can both be used for screening; the method does not appear to affect intervention effectiveness. Screening and recruitment is time-consuming, especially when targeting lower socioeconomic status and age under 45 years. The intervention intensity is more important for effectiveness than the mode of delivery. Moderate changes in several lifestyle habits lead to good intervention results. A minimum of 3-year follow-up seemed to be required to show a reduction in diabetes risk in high-risk individuals. In participants < 45 years, the achieved results in outcomes were less pronounced. The complementary review included 12 studies, with similar results regarding intervention targets and delivery modes, as well as clinical significance.

**Conclusion:**

This narrative review highlighted several important aspects that subsequently guided the development of the Feel4Diabetes high-risk intervention. Research on diabetes prevention interventions targeted at younger adults or vulnerable population groups is still relatively scarce. Feel4Diabetes is a good example of a project aiming to fill this research gap.

**Trial registration:**

clinicaltrials.gov NCT02393872, registered 20th March 2015.

## Background

The number of people with type 2 diabetes is reaching epidemic proportions all over the world [1]. In Western societies, a social gradient in the prevalence of type 2 diabetes and its risk factors is also well-documented, vulnerable population groups having greater burden than those in higher social strata [2–4]. The clinical manifestation of type 2 diabetes usually appears later in life, but many of the risk factors and behaviours develop much earlier and many disparities in health are rooted already early in life. The prevalence of the disease is growing also in younger individuals as a result of increasing obesity rates, unhealthy diet and physical inactivity already present during childhood [1].

Tackling the type 2 diabetes epidemic is a major public health challenge. Most of the diabetes prevention interventions so far have been targeted at middle-aged people who already have non-diabetic hyperglycaemia [5–7]. These interventions are highly warranted, as identified high-risk individuals cannot be left untreated and the achieved risk reduction has been shown to be most pronounced among individuals who are already close to the diagnostic limit [8]. However, to achieve largest impact on population level, prevention emphasizing healthy lifestyle should be started already during childhood and continued throughout the life course. A new challenge is to learn from the previous type 2 diabetes interventions and tailor them for younger individuals who have traditionally been considered as low risk and who therefore have not received the appropriate attention [9].

The objective of the Feel4Diabetes project was to develop, implement and evaluate a school-, community- and family-based intervention program for the prevention of type 2 diabetes among vulnerable families with children in primary school, in six European countries during 2015–2019 [10]. The 2-year intervention included two components: “all families component” and “high-risk families component”. The all families component was targeted at school-aged children and their families and tailored to improve the diet, physical activity patterns, and body weight according to national guidelines. A school setting was chosen to reach families with different socioeconomic backgrounds and to utilize the school as an intervention venue. Feel4Diabetes also focused on areas with lower socioeconomic status to reach the most vulnerable populations for type 2 diabetes.

The parents of the participating families filled FINDRISC-questionnaire and the parents with high risk scores were invited to participate in type 2 diabetes prevention study, e.g. the high-risk families component. These parents got in addition to school based activities more intensive intervention, including individual and group sessions about type 2 diabetes, healthy eating and exercising following a SMS intervention based on tips and reminds about healthy lifestyle. Feel4Diabetes-study was registered in clinicaltrials.gov with registration number NCT02393872.

As part of the PRECEDE phase of the PRECEDE-PROCEED model of Feel4Diabetes [11] several literature searches were completed, to guide the development of evidence-based implementation of the Feel4Diabetes intervention. In addition to this review focusing on adults, a review focusing on studies implemented in school setting aiming to enhance healthy lifestyle in children was conducted [12]. The aim of the work presented in this paper was to systematically review the available research literature on type 2 diabetes prevention strategies targeted at adult high-risk individuals and find state-of-the-art methods in all phases from risk identification to implementation and maintenance to use in Feel4Diabetes high-risk families component. The primary literature search was conducted in 2015 before beginning of the Feel4Diabetes-study and updated in 2019 to provide a comprehensive review of the subject. Specifically, our aim was to pinpoint effective type 2 diabetes prevention strategies regarding vulnerable population groups, as well as strategies that have been successfully implemented among under middle-aged population groups.

## Methods

### Search strategy

For the primary literature search conducted in 2015 a search strategy was developed in consultation with an information specialist. The information specialist completed three searches using search terms related to ‘diabetes’, ‘prevention’, ‘intervention’ and ‘efficacy’. The first search was a general search for diabetes prevention interventions from scientific literature in OvidSP (MEDLINE), Web of Science, EBSCOhost and Cochrane databases. The second search was targeted to interventions on vulnerable populations and in addition to search terms used in search one, the term ‘vulnerability’ and related terms were used. The third literature search was completed to find grey literature using Open Grey, Greylit -reports, NICE Evidence Search and Google search engines with same search terms as in the first one. The question about identification of high risk adults was explored in the context of preventive interventions. The information specialist did preliminary selection according to the search strategy. Two reviewers independently examined titles and abstracts and selected relevant articles according to inclusion criteria. In case of disagreement, inclusion was resolved through discussion.

The complementary literature search was done in 2019 to update the original work with the most recent type 2 diabetes prevention studies. The search terms used were the same as in first search for the primary literature review and the search was done using PubMed (MEDLINE). The complementary search was done by one reviewer and the search was complemented using cross-references in the already included publications and reviews to ensure coverage.

### Inclusion and exclusion criteria

Inclusion criteria based on title and abstract were:

1. Type of study: Randomized controlled studies (RCT) or pre-post intervention studies that considered the effectiveness of a lifestyle intervention (diet and/or exercise).
2. The stated aim of the study: type 2 diabetes risk reduction or prevention of type 2 diabetes.
3. Population: Adults (18 years and over) identified as being at high risk of developing type 2 diabetes identified having prevalent risk factors (for example obesity, sedentary lifestyle, family history of diabetes, metabolic syndrome, impaired glucose tolerance (IGT), impaired fasting glucose (IFG), prediabetes, hyperlipidemia, gestational diabetes, cardiovascular disease, elevated diabetes risk score or elevated cardiovascular risk score).
4. Outcome of the study: Development of diabetes or change in diabetes risk, measured by a reliable and scientifically approved risk marker like weight, body mass index (BMI), fasting glucose or glucose tolerance.
5. Study published: in the English language and as full-length articles between January 1st, 2000 and January 29th, 2015 in primary search OR between January 29th, 2015 and February 28th 2019 in complementary search
6. Follow-up time of at least 12 months OR at least 6 months if median/mean age of participants was < 45 years

After the selection of relevant publications based on abstracts (*n* = 232 in the primary search), the publications originating from the same study were combined, the full-length papers were acquired and read. Studies were excluded if they did not meet the inclusion (if the study was not RCT or pre-post study, the aim was not to prevent type 2 diabetes, participants inclusion was not based on type 2 diabetes risk, the outcome was not a measured risk marker for type 2 diabetes or the article was not in English and published before January 2000) criteria or the study population included a large proportion of people with diabetes (over one fourth), the results of the primary endpoints were not published or follow-up time was less than 6 months. Originally, we decided to exclude studies with less than 12 months follow-up time, to emphasize the evidence on long-term effectiveness of the intervention. However, as the research including younger participants (< 45 years of age) proved to be scarce, we modified the criteria to include studies with at least 6 months follow-up time if they included participants within the age range of 18 to 45 years.

### Data synthesis

**Fig. 1:**
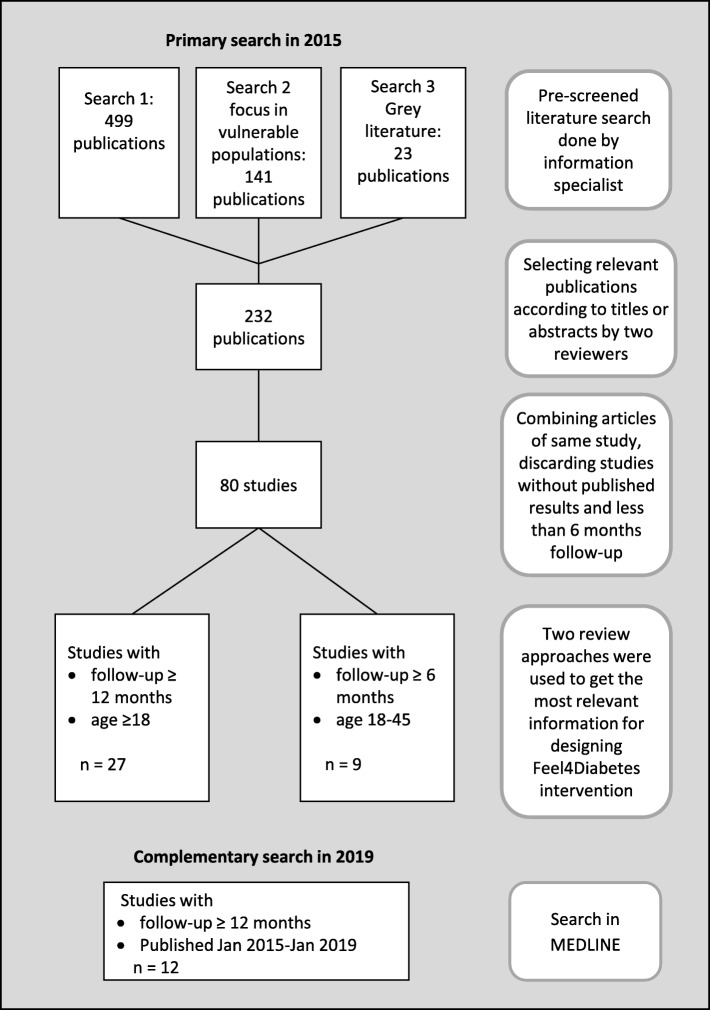
Flow chart of the literature review process

The selected publications (*n* = 80) in the primary search showed that the majority of the published diabetes prevention studies have been targeted at older population groups than the target group of the Feel4Diabetes intervention (parents with school-aged children). It is known that increasing age is a significant risk factor for type 2 diabetes and the study by Deeks et al. [13] found age dependent differences in health beliefs and screening participation rates. Older people were more likely to participate in specific health checks including blood glucose and cholesterol measurement than younger people. Presumably older people have different life circumstances and thus different barriers for participation and changing lifestyles compared with younger ones. Relying on studies with mainly older participants (as those with sole number override the studies on younger people) might have steered the conclusions off target. Therefore, two different review approaches were conducted. In first approach the studies with the participants aged ≥18 years and minimum follow-up of 12 months (*n* = 27) were reviewed. In second approach the studies with mean or median age of participants less than 45 years and minimum follow-up of 6 months (*n* = 9) were included. In the complementary search all studies had follow-up time over 12 months (*n* = 10). A flow chart of the selection of relevant studies is presented in Fig. 1.

To present the findings from the literature reviews in a systematic way, we used the Reach, Effectiveness, Adoption, Implementation, and Maintenance (RE-AIM) framework designed for assessing interventions and public health programs [14]. The overall goal of the RE-AIM framework is to encourage implementers to pay more attention to core elements, which can improve adoption and implementation interventions.

The summary tables of the selected studies (see Tables 1, 2 and 3) were prepared and reviewers independently evaluated the clinical significance of the results presented for each study, to facilitate interpretation of the effectiveness versus the design, methods, delivery, and costs of intervention. The clinical significance of the study results was scored as follows: meaningful reduction in diabetes risk; meaningful improvement in (most) target risk factors; meaningful improvement in some/few risk factors; or no effect.

**Table 1:** Ov**e**rview of the studies targeting participants aged ≥ 18 years and minimum follow-up of 12 months

	Reach		Implementation & adaptation		Efficacy & maintenance
Name of study, Acronym Country References	Target group, Inclusion criteria	Screening, recruitment, study population (n, sex, mean age), drop-outs	Study design, follow-up (FU) duration, lifestyle goals/targets	Intervention delivery, intervention duration, change theories	Results Clinical significance estimate*
Daqing Diabetes Prevention Study China Pan et al., 1997 [5] Li et al., 2008 [15]	People living in Daqing area, > 18 y old IGT	110,660 people screened with OGTT 577 randomized sex: 54% men age: 45 ± 9 y	Cluster randomized controlled study in four groups: control, diet only, physical activity (PA) only, or diet plus PA FU 6 years (*n* = 533) + 20 years. Goals: Diet (increase vegetables, decrease alcohol and sugar, caloric and weight reduction if overweight), PA (1–2 units/day; unit = 30 min of slow walking etc., 20 min of fast walking etc. or 5 min of jumping rope etc.) or combined both	Individual counselling + compliance evaluation by physician/nurse every 3 months + small groups weekly for 1 month, monthly for 3 months and every 3 months thereafter. Intervention duration 6 years.	HRs (adjusted for baseline BMI and f-Glu): HR = 0.69 for diet vs CG, *p* < 0.03; HR = 0.54 for diet + PA, *p* < 0.0005; 0.58 (diet+PA), *p* < 0.005; HR = 0.41 for PA vs CG *p* < 0.0005. The average number of PA units per day was significantly higher after 6 years. No significant changes in diet. ++
Diabetes Prevention Study, DPS Finland Tuomilehto et al., 2001 [6] Lindström et al., 2003 [16] Lindström et al., 2006 [17] Lindström et al., 2013 [18] Wikström et al., 2009 [19]	People with high risk for T2D IGT in two OGTTs IGT (according WHO 1980) BMI > 25 kg/m^2^ age 40–64	Opportunistic screening based on age and BMI; previous study patient files; newspaper ads. Stepwise screening (1st OGTT - > 2nd OGTT). Approximately 10% of those tested were eligible. 522 randomized sex: 33% men age: 55 years	RCT in 5 study centres in Finland. Intensive, individualized intervention vs. general “mini-intervention” at baseline. FU mean 3.2 years (*n* = 482) + 7 years + 13 years Goals: < 30% of total energy from fat; < 10% energy from saturated fat; at least 15 g of fiber/1000 kcal; at least 4 h/week physical activity; > 5% weight reduction. PA sessions (2/week in free gym) were offered.	7 individual counselling sessions with nutritionist (at 2w, 5w, 3 m, 4 m, 6 m (first 1 h, later 30 min), every 3 months thereafter. Sessions included pre-defined topic + review of food and exercise diaries + goal setting with participants. 1 year intensive phase plus maintenance 1 to 5 years, mean duration 4 years. Optional very low caloric diet phase. Stages of change model, emphasizing self-efficacy, monitoring, feedback, behaviour planning, relapse management.	Diabetes incidence in 3.2 year (main results) reduced by 58%.Significant difference in changes of weight (− 4.5 kg in IG vs. -1 kg in CG at year 1), BMI, waist circumference, diet, physical activity, fasting and 2 h glucose in IG compared to CG. Effects of intervention sustained after 7 years and after 13 years. ++
Diabetes Prevention Program, DPP USA Knowler et al., 2002 [7] Rubin et al., 2002 [20] Fujimoto et al., 2000 [21]	aiming for 50% ethnic minorities IGT (WHO 1980) fasting glucose > 5.5 mmol/l age > 25 y, BMI > 24 kg/m^2^	Recruitment with mail, advertisements in media and work sites. 133,683 individuals pre-screened, 26,518 screened with an OGTT. 3.048 randomized sex: 33% men age: 50 + 11 years 45% from ethnic minority groups	RCT in 27 clinical centres Intensive lifestyle vs. metformin vs. placebo FU mean 2.8 years, drop-out 7.5% Weight reduction 7% Diet: fat 25 E% Physical activity (e.g. brisk walking) 150 min/week (700 kcal/week)	Main goal to achieve and maintain a weight reduction of > 7% through a healthy low-calorie, low-fat diet and to engage in physical activity of moderate intensity. Goal-based behavioural intervention lasting for 2.8 years (mean); case-managers (1 per 20–26 participants) held 16-session core curriculum in groups during the first 24 weeks; individual session monthly	T2D risk reduction 58% in lifestyle vs placebo after 2.8 years. 50% of IG met the weight reduction goal after 24 weeks. Mean weight loss 7 kg (7%) at 1 year. ++
Prevention of type 2 diabetes by lifestyle intervention: a Japanese trial in IGT males Japan Kosaka et al., 2005 [22]	30–60 year old men IGT (according WHO 1980)	Random selection of men with IGT from health-screening program for mostly government employees. 458 randomised *n* = 356 in CG, *n* = 102 in IG sex: 100% men age: mean NA	RCT intensive care vs. standard hospital care (1:4). FU 4 years. Drop-outs 5.6% in CG, 4,7% in IG BMI < 22; reduce energy intake by 10%; increase vegetable intake; fat < 50 g/day; alcohol < 50 g/day; eating out once /day or less; walking 30–40 min / day	Face-to-face counselling by nurse in hospital every 2–3 months, 4-year intervention. Regular weight self-monitoring. Concrete, standardised advices to reach the goals of the study.	Relative risk reduction 67% in 4 years. Body weight reduction 2.2 kg at 4 years vs. 0.39 kg in the control group ++
Indian Diabetes Prevention Programme, IDPP-1 India Ramachandran et al., 2006 [23]	Middle-class population; 35–55 years; IGT in two OGTT (WHO 1999)	Recruiting by workplace announcements and circulars. 10,839 subjects underwent initial screening using glucometer. *n* = 531; sex: 81% men age: 45.9 years	RCT in community-based setting in 4 groups: Control; metformin (MET); lifestyle modification (LSM); LSM + MET. FU 3 years (*n* = 502). Goals: > 30 min brisk walking daily: reduction in total calories, refined carbohydrates and fat; avoidance of sugar and inclusion of fibre-rich foods	Participants had a personal session at 6-monthly intervals and were contacted by phone every month. Diet modification was advised for each subject.	Absolute risk reduction at year 3 was 15.7% in LSM, 14,5% in MET and 15.5% in LSM + MET compared to control (all *p*-values for HR < 0.03). ++
N/A UK, Newcastle Oldroyd et al., 2006 [24] Oldroyd et al., 2001 [25]	European origin men and women aged 24–75 years IGT in 2 OGTT (WHO 1985)	Recruited people from previous research studies, local hospital and GP databases. *n* = 78 sex: 57% men age: 58.2 years	RCT in hospital setting; control vs intervention FU 2 years (*n* = 54) BMI < 25 kg/m^2^, dietary fat < 30 E%, polyunsaturated to saturated fat (P:S) ratio ≥ 1.0; carbohydrate 50 E%, dietary fibre > 20 g per 4.2 MJ, 20–30 min aerobic exercise at least once a week	Individual counselling from a dietician and physiotherapist using the stages of change model. Intervention lasted 2 years and 12 sessions with duration of 15–20 min.	Meeting intervention goals was higher in IG for all other but P:S ratio. In IG weight change was − 1.8 kg vs + 1. 5 kg in CG at 24 months. Significant change in fasting serum insulin between groups at 12 months. +
Hoorn Prevention Study The Netherlands Lakerveld et al., 2012 [26] Lakerveld et al., 2013 [27]	Age 30–50 At least 10% risk for T2D and/or CVD estimated according formula of ARIC and SCORE projects.	A screening invitation to GP customers (*n* = 8193). 2401 respond, 921 eligible based on waist circumference. 772 were screened. *n* = 622 sex: 42% men age: 43.5 years	RCT in general practice. FU 12 months (*n* = 502). Goals were at least one fruit, at least 200 g vegetables and at least 30 min PA per day.	Participants were offered 6 face-to-face sessions (30 min) and 3 monthly telephone sessions with trained nurses. Methods were based on motivational interviewing, problem solving treatment, theory of planned behaviour and theory of self-regulation. CG got brochures of health guidelines.	No significant results in weight or fasting glucose or glucose tolerance. Increase in fruit intake between baseline and 6 months (1.1 - > 1.3 pieces per day) but not after 12 months. Median participation in sessions was 2. (−)
The Joetsu Diabetes Prevention Trial Japan Kawahara et al., 2008 [28]	20–70 year old men and women IGT (ADA 2003)	Recruiting from 11 outpatient practices and health evaluation and promotion centres in Joetsu area. Patients with BMI 20–34 kg/m^2^ and FPG ≥ 5.6 mmol/l or HbA1C 5.2–6.4% were screened *n* = 426 sex: 47% men age: 51.4 years	Community-based clinical trial comparing short-term hospital (STH, *n* = 143) or outpatient diabetes education support (DES, *n* = 141) to no-treatment (*n* = 142). Mean FU 3.1 years. Goals for STH were 25–30 kcal / ideal body weight kg / day; 20–25% E% of fat; at least 30 min/day walking or exercise at least 5 times a week. Goals for DES were to follow diabetes guidebook.	STH group had 2-day hospital stay with a course of nine group/individual lessons covering diet, exercise and behaviour modification. Subsequent sessions every 3 months were offered individually. DES group got written information and 3-monthly individual sessions of a healthy lifestyle. Lessons were 20–40 min and were taught by different medical specialists. Mean intervention duration was 3 years.	The incidence of diabetes was 42 and 27% lower in STH and DES groups compared with no-treatment group, and 21% lower in STH than DES. Also FPG, 2 h OGTT plasma glucose, HbA1C and weight changes between groups were significantly different. STH) was more cost effective than DES ++
EDIPS-Newcastle UK Penn et al., 2009 [29]	> 40 years, BMI > 25 kg/m^2^, IGT in two OGTTs	Recruiting by invitation letter to eligible customers of primary care physicians. 1567 were contacted; 1084 replied; 682 agreed to testing; 482 completed at least one OGTT *n* = 102 sex: 41% men age: 57.1 years	RCT of two arms; intervention vs usual care. Mean FU 3.1 years (n = 42), up to 5 years > 50 E% carbohydrate; < 30 E% fat; reduce saturated fat intake; increase fiber intake; BMI < 25 kg/m2	Approx. 24 sessions with dietitian and physiotherapist as individual motivational interviewing for behavioural changes, including feedback from food diaries, weight and waist measurements. Cooking groups and discount of leisure service card was offered. Quarterly newsletter containing recipes, nutritional info and exercise suggestion was sent. Control group got usual care.	Weight reduction was 2.3 kg in IG vs. no change in CG, *p* = 0.007 at year 1 but no significant difference in consecutive years. The overall incidence of diabetes was non-significantly reduced by 55% in the intervention-group vs. the control group, with RR 0.45 (95% CI 0.2 to 1.2). (+)
PREDIAS Germany Kulzer et al., 2009 [30]	20–70 years BMI ≥ 26 IGT or IFG	Invitations by primary care physician based on FINDRISC > 10. *n* = 182 sex: 57% men age: 56.3	Two armed randomized control study in general practice. FU 12 months (*n* = 165). Goals based on DPP.	12 × 90-min group lessons in 12 months. Program was based on self-management theory and delivered by diabetes educator or psychologist. Participants got an exercise book, with diabetes prevention information and worksheets for lessons. Control group got written information about diabetes prevention.	There was significant difference in weight loss (−3.8 ± 5.2 vs. − 1.4 ± 4.09 kg), reduction in fasting glucose, increase in duration of PA and changes in eating behaviour after 12 months between intervention and control group. +
Telephone support in addition to Greater Green Triangle Diabetes Prevention Program Australia Dunbar et al., 2010 [31]	40–75 years FINDRISC > 12 points	Participants who completed the 12 month diabetes prevention program and were willing to participate in follow-up. *n* = 205 sex: 28% men age: 56.6 years (self-care); 57.1 (telephone support)	Telephone support vs self-care after 1 year lifestyle intervention pretest-posttest study for next 18 months FU 30 months (*n* = 164) < 30 E% fat; < 10 E% saturated fat; > 15 g fibre/1000 kcal; > 4 h/week moderate PA; > 5% weight reduction	Telephone support started after 12 month original intervention consisting of 6 group sessions. Telephone group got up to 12 calls following semi-structured interview with questions regarding personal goals.	There wasn’t significant difference between telephone support and self-care group. Original interventions improvements in 12 months were generally maintained to 30 months in both groups. (−) for telephone support
DE-PLAN, Greece Greece Makrilakis et al., 2010 [32]	FINDRISC ≥15 points	3240 individuals were screened with FINDRISC-questionnaires; 620 were eligible. *n* = 191 sex: 40% age: 56.3 y	Pretest-posttest study in community setting in Greece. FU 12 months (*n* = 125) Reduce saturated and trans fat, sugars and sweets, refined cereals; ≥ 5 portions of fruits and vegetables per day; PA ≥ least 30 min 5 times a week. Weight reduction was recommended if overweight.	A dietitian held 6 group intervention sessions during 1 year. Each session was focused on one of goals and included information, discussion and written material. Sessions were held in workplaces or near participants’ residence. Evaluation of achieving goals was discussed in the beginning of each session.	Weight, BMI, blood pressure, and total cholesterol reduced significantly in those who completed program. Also reduction in whole fat dairies, processed meats, sugars and refined cereals was significant. +
PREDIMED-Reus Spain Salas-Salvadó et al., 2011 [33] Martínez-González et al., 2012 [34]	Men 55–80 years; Women 60–80 years; At least 3 risk factors for cardiovascular disease: smoking; hypertension; dyslipidemia; BMI > 25 kg/m^2^; family history of cardiovascular disease	1125 participants were screened in primary care centre and 870 fulfilled inclusion criteria. Of these 452 were diabetics, so 418 were the final population in this sub-study. *n* = 418 sex: 42% men age: 67 y	RCT for primary cardiovascular prevention in three arms: “low-fat” –control; Mediterranean Diet (MedDiet) + oil; MedDiet + nuts. Median FU 4 years. MedDiet; use olive oil abundantly; increase consumption of fruits, vegetables, legumes, nuts and tomato sauce for cooking; reduce total and red meat use and use fish and white meat instead; avoidance of butter, cream, fast food, sweets, pastries and sugar-sweetened beverages; moderate use of red wine. Control: reduce all type of fat, no free foods. All groups: No energy restriction, no PA promoted	Dietitians gave personalized dietary advice to participants on basis of a 14-item (MedDiet) or 9- item questionnaire (control). At inclusion and quarterly there after dietitians administered individual and group sessions. Participants were offered written material including descriptions of seasonal foods, shopping lists, weekly meal plans and cooking recipes. In addition participants in MedDiet groups were given free virgin olive oil (1 l/week) or nuts (30 g/day).	Hazard ratio for diabetes was 0.55 (0.32–0.95 95%CI) for both MedDiets compared with control diet in crude model and 0.48 (0.27–0.86) in multivariate adjusted model. Diabetes incidence was lower in participants who complied with the MedDiet better. Largest risk reduction was seen in MedDiet in subgroups of women vs men, over 67 year-old vs under and with those whose fasting glucose > 6.1 mmol/l in baseline than those who had ≤6.1 mmol/l. ++
DE-PLAN-Krakow Poland Gilis-Januszewska et al., 2011 [35]	FINDRISC points > 14; OGTT to exclude diabetics	Recruiting from primary health care centres. 566 completed questionnaire; 368 eligible; 275 underwent OGTT *n* = 186 sex: 22% men age: 56.1 y	Pretest-posttest study in primary health care in Poland FU 12 months (*n* = 175) Goals were weight loss, reduced intake of total and saturated fats, increased consumption of fruits, vegetables and fibre and increased PA.	Active phase of intervention consisted of 10 group sessions in first 4 months followed by 6-month maintenance with six motivational phone calls and two motivational letters. PA sessions were offered once or twice a week. Social and family involvement was encouraged. Prevention managers were educated.	Significant changes in weight, BMI and waist circumference. No significant changes in fasting glucose or glucose tolerance test results. +
Making the Connection Healthy Living Program, MTC HLP USA Ruggiero et al., 2011 [36]	underserved latino population; age 18–65 years; BMI > 24.9, normal glucose or prediabetes; Latin background	Community-based health screening events (schools, family centers, hospital etc). 1162 screened, 367 tested for eligibility, 244 eligible. *n* = 69 sex: 7% men age: 38 y	Single-group, non-randomized follow-up, community-based translation of DPP. FU 12 months (*n* = 57). DPP goals: Weight reduction 7%; Diet: fat 25 E% Physical activity (e.g. brisk walking) 150 min/week	Culturally specific intervention was developed and conducted in collaboration with the community to minimize barriers to participation education, literacy, language, income, transportation, lack of medical coverage. 22 group sessions during 1 year, delivered by trained community health workers. Cook books, pedometer, scales provided. Group walks arranged. Participants attended 57% of group sessions.	At 6 m, 20% achieved 7% weight reduction, and at 12 m 16% achieved. Moderate improvements in body weight (− 4.8 kg at 6 m, − 2.8 at 12 m), waist, fruit and vegetables, fat intake, PA were observed at 6 m but attenuated at 12 m. Forward movement in “stages of change” scale was observed. +
The Prevention of Diabetes and Obesity in South Asians: PODOSA UK Bhopal et al., 2014 [37] Douglas et al., 2013 [38] Douglas et al., 2011 [39]	South Asian families living in UK > 35 years; IGT and/or IFG	Recruiting via National health service, South Asian organizations and peer to peer. Pre-screening by waist circumference. 1319 screened with an OGTT, 196 (15%) were eligible. *n* = 171 (156 families) sex: 45.6% men age = 52.5 y	Family-based two armed RCT in South Asians living in UK FU 3 years Goals were calorie deficit diet, at least 30 min brisk walking per day and at least 2.5 kg weight reduction.	Intervention visits were done in participants’ homes, 15 for intervention group and 4 for control group in 3 years. Practical dietary counselling: cooking, food shopping, food labelling and recipes. Counterweight program was used to dietary counselling. Change management tools, self-reflection and cultural adaptations were included.	Weight change in the IG was − 1.13 kg (SD 4.12) and in CG + 0.51 kg (SD 3.65), an adjusted mean difference of − 1.64 kg (95% CI − 2.83 to − 0.44).No difference in changes of fasting plasma glucose, OGTT or physical activity. Progression to diabetes was observed less frequently in the IG than the CG (OR 0·68) but not statistically significant (*p* = 0·3705). (+)
Zensharen Study for Prevention of Lifestyle Diseases Japan Saito et al. 2011 [40]	30–60 years; BMI > 24 IFG	First screening from health check-ups and eligible people were invited to OGTT (diabetics were excluded). *n* = 641 sex: 78% men age: median 50 y in IG, 48 y in CG	Unmasked, multicenter RCT in health care setting: frequent intervention group (FINT *n* = 311) vs. control (*n* = 330). FU 36 months (*n* = 562). Goals: 5% weight loss; reduce total energy intake; fat 20–25 E%; carbohydrate 55–60 E%; increase fibre intake and moderate alcohol intake. Increase of incidental PA to 200 kcal/d.	FINT got individual instructions and follow-up support for lifestyle modification from mainly dieticians and nurses at least 9 times. Self-monitoring using pedometers and body weight recording sheets. Control group got similar individual instruction 4 times during first 12-months without follow-up support or self-monitoring tools.	The HR for T2D in FINT was 0.56 (95% CI 0,36-0,87) compared to control. In FINT IFG only group HR was 1.17 (0.50–2.74), and in FINT IFG + IGT 0.41 (0.24–0.69), compared to corresponding CGs. > 5% weight loss was significantly more achieved in FINT group during the first 12 months. Also PA goals and reducing energy intake goal were achieved more in FINT group. ++
APHRODITE The Netherlands Vermunt et al., 2011 [41] Vermunt et al., 2012 [42] Vermunt et al., 2012 [43]	FINDRISC ≥13; age 40–70 years	FINDRISC-questionnaire was sent to GP patients from 14 primary care practices (*n* = 16,032). Individuals with a score ≥ 13 were invited (*n* = 1533) for OGTT and diabetics were excluded. *n* = 925 sex: 38% men age: 58 y	RCT in Dutch primary care (IG *n* = 479, CG *n* = 446) FU 2.5 years (IG *n* = 368, CG *n* = 341) Weight reduction ≥5% if overweight, PA for at least 30 min a day / 5 days a week, fat intake < 30 E% and saturated fat < 10 E%, dietary fibre intake ≥3.4 g per MJ	Participants were offered 11 individual sessions with nurse or general practitioner, one with dietitian and 5 group sessions with dietitian and physiotherapist. Intervention lasted 30 months. Dietary advices were based on food diary. The intervention was based on trans-theoretical model (the stages of change).	Differences between groups were significant only for total physical activity and saturated fat and fibre intake. In the intervention group, self-efficacy was significantly higher in individuals successful at losing weight compared with unsuccessful individuals. (+)
Lawrence Latino Diabetes Prevention Project (LLDPP) US, Massachusets Ockene et al., 2012 [44]	Low-income latinos 25–79 y old latino / hispanic ethnicity; ≥ 25 y old; BMI > 24; > 30% increased according T2D risk algorithm	Recruiting (*n* = 949) from local health centre and local media. *n* = 312 FU: 289 (CG 142, IG 147) sex: 25.6% men age: 52 y	Randomised community-based, culturally tailored, literacy sensitive lifestyle intervention (*n* = 162) vs. usual care (*n* = 150). FU 1 year (*n* = 289) Increase intake of whole grains and vegetables; reduce sodium, total and saturated fat, portion sizes and intake of refined carbohydrates and starches; increases walking by 4000 steps/day	3 individual sessions at home (1 × 1 h, 2 × 0.5 h) and 13 group sessions (1 × 1.5 h, 12 × 1 h) over 12-month period. Participants got cash incentives at baseline, at 6- and 12-months. Participation was maximized with compensatory sessions and home visits. Practical, hands-on methods and demonstrations were used. Intervention was based on social cognitive theory and patient-centred counselling.	Participants lost more weight in IG (−2.5 lb) than in CG (+ 0.63 lb), effect of intervention − 2,5 lb. (*p* = 0.004). Also HbA1c reduced more in IG vs CG (effect of intervention − 0.10%, *p* = 0.009). Participants in IG reduced more energy intake from fat and saturated fat and increased dietary fibre intake. +
E-LITE US Xiao et al., 2013 [45] Ma et al., 2013 [46]	≥ 18 years BMI ≥ 25; IFG or metabolic syndrome (2005 AHA)	Recruiting from a primary care clinic: 3439 contacted, 752 screened BL *n* = 241 (81 cont; 79 coach-led; 81 self-directed) sex: 53% men age: 52.9	RCT in primary care with three arms: control, coach-led group intervention vs. self-directed DVD intervention. FU 15 months (*n* = 221) Weight loss goal based on DPP	Intensive intervention (3 months) included 12-session in groups or at home via DVD. Coach-led group had food tasting and guided PA and self-directed group got one face-to-face session, weight scale, pedometers and biweekly reminder e-mails during 12 month maintenance phase. All intervention participants were trained to use self-monitoring web portal.	At month 24 the mean change in BMI from baseline was −1.9 +/− 0.3 kg for coach-led group (p = 0,001 vs CG); − 1.6 +/− 0.3 kg for self-directed group (*p* = 0.003) and − 0.9 +/− 0.3 in the control group. Fasting plasma glucose was significantly more improved in IGs compared to CG. +
RCT of SMS for Drivers With Pre-DM China Wong et al., 2013 [47]	Professional drivers IFG/IGT; had a mobile phone	Screened 3376 drivers identified by community screening and media advertisement. n = 104 subjects age: 53 y sex: 93.3% men	RCT of short message service (SMS) intervention on vs. control (leaflet) FU 24 months (IG *n* = 41;CG *n* = 29). “Diabetes-related information in reducing the risk of developing diabetes”	Participants got sms from 4 themes: diabetes information; lifestyle change; how others would appreciate the lifestyle modification; self-efficacy enhancing statements. In the first 3 months sms were sent 3 times a week, next 3 months weekly and last 18 months monthly. Both groups got leaflets about diabetes. Intervention was based on theory of planned behaviour and social cognitive theory.	No significant reduction in diabetes risk after 12 or 24 months. Significant mean differences in diastolic blood pressure and HDL-cholesterol over time between the groups. Intervention cost was 5.05 $/ subject (−)
DH!AAN The Netherlands Admiraal et al., 2013 [48] Vlaar et al., 2012 [49]	South Asian migrants Age 18–60 y; IFG, IGT, HbA1c > 6.0% or HOMA-IR > 2,39	2307 screened via general practices (invitation letter with reply card), followed by reminder and telephone call). *n* = 536 age: 44.9 y sex: 49.4% men	RCT in general practice among South Asian migrants in Netherlands getting a culturally targeted intervention or generic lifestyle advice (control). FU 2 years (*n* = 335). Goals according to SLIM study; based on current guidelines on diet and physical activity.	6–8 individual sessions in general practice during 6 months, 2 sessions during the next 6 months + 1 family session + two cooking classes. 20-week supervised exercise program was offered. Trained dieticians gave dietary counselling using motivational interviewing. Participants got a gift coupon for participating in baseline measurements. Control arm got 2 group sessions + 2 flyers.	No significant results. Median participation in 5 individual sessions. High drop-out and low participation 26% participated in family session, 26% in cooking sessions and 22% in PA sessions. (−)
“Group Lifestyle Balance™ program in the community setting USA Kramer et al. 2014 [50]	26–80 years old BMI ≥ 25 kg/m^2^; IFG / metabolic syndrome; ≥ 25 years old	Recruiting via GPs, information letters, e-mail contact and newspaper advertisement. *n* = 81 sex: 22% men age: 52.9 y	Group Lifestyle Balance (GLB) program in three outpatient-hospital in a pretest-posttest setting. FU 12 months (*n* = 52) 7% weight loss; 150 min PA/week	12-session group lifestyle intervention adapted from DPP delivered by trained diabetes educators in groups over 12 to 14 weeks and monthly thereafter. Each session lasted 1 h. Handouts, self-monitoring booklets, fat- and calorie-tracking book and a pedometer were given to participants.	Significant changes: weight loss was 5.1% (*p* < 0,001); decrease in waist circumference; fasting plasma glucose; LDL-cholesterol; triglycerides and blood pressure. +
Subanalysis of the Japan Diabetes Prevention Program Japan Sakane et al., 2014 [51]	30–60 years IGT	People measured in yearly health check-ups were recruited using posters, fliers and by word of mouth. 1279 were screened with OGTT. *n* = 304 sex: 50,0% men age: 59% men	RCT in 32 primary health care centres using existing resources in two arms. mean FU 2.3 years (*n* = 213) 5% reduction in body weight in overweight and obese; increase energy expenditure by 700 kcal per week; < 25 E% fat; < 160 kcal/day from alcohol	Study nurse held four 2–3-h group sessions during first 6 months followed by biannual 20–40 min individual sessions, intervention was 3 years. After first year individual sessions were held on phone. Personalised goals were set. A booklet was given and monthly tip cartoons were sent via fax. Self-efficacy, self-monitoring and trans-theoretical model was used.	No significant results in T2D risk. In IG participants with BL HbA1c levels ≥5.7% (*n* = 177) cumulative incidence was significantly lower. Significant results: at 1 year IG had improved body weight and daily non-exercise leisure time energy expenditure and at 3 years better Matsutada index. (+)
Use of Information Technology in the Prevention of Diabetes India Nanditha et al., 2014 [52] Ramachandran et al., 2013 [53]	Men No major illness; age 35–55 years; positive family history of T2D; BMI > 23; IGT	First screening with questionnaire (*n* = 8741) and then OGTT first with a glucometer and confirmatory venous blood glucose within 1 week. *n* = 537 sex: 100% men age: 46 y	RCT in industrial male workers lifestyle modification with SMS. FU 2 years (*n* = 517) Avoidance of simple sugars and refined carbohydrates; total fat intake < 20 g/day; restrict use of saturated fat; increase fibre; enhance aerobic exercise > 30 min brisk walk/day; walk 3–4 km in 30 min at least 5 days a week	All participants got personalised lifestyle modification in the beginning. SMS-group received mobile phone messages at frequent intervals (2–4 sms/week). SMS content was tailored according to participant’s stage of change in trans-theoretical model.	Risk reduction in sms-group compared to control was 9% (HR = 0.64 CI 0.446–0.917 *p* = 0.015). ++
“Lifestyle modifications in Chinese women who had gestational diabetes mellitus” China Shek et al., 2014 [54]	Age > 18 years; GDM history; IGT 6–8 weeks after delivery; excluded if insulin for GDM	Patients from hospitals were invited if criteria were fulfilled *n* = 450; sex: 100% women age: 39 y	RCT conducted in hospital in Hong Kong. Intensive lifestyle intervention vs. no intervention (control). FU 36 months (*n* = 423). Optimal caloric intake (based on Harris-Benedict) for ideal body weight	7 individual sessions in 3 years (3 m, 6 m 12 m, 18 m, 24 m 30 m, 36 m). Dietician and study nurse gave individual dietary and exercise advice based on food and exercise records (*n* = 7).	No significant difference in cumulative incidence of diabetes. In women > 40 years, difference between groups was significant. Significant differences between groups at 1 year in BMI and waist-hip ratios, but not significant in the end of the study. (+)
“Prevention of diabetes in Finnish airline” Finland Viitasalo et al., 2012 [55] Viitasalo et al., 2015 [56]	Airline workers (majority shift-work) FINDRISC > = 10 or IFG or IGT	Occupational health care check-up *n* = 2312, 657 had high risk and were offered intervention. *n* = 350 sex: 60% men age: 47 y	Work-site study targeted at identified high-risk workers of an airline. Average FU 2.5 years (*n* = 402). DPS goals and other goals according to risk factor levels (BP, cholesterol).	1–3 individual lifestyle counselling sessions in addition to the check-up by nurse/physician. Lifestyle sessions were delivered by diabetes nurse or nutritionist.	Among elevated risk men, body weight was slightly reduced and 14.3% lost > 5% of weight, and cholesterol and LDL decreased. Those men who attended more lifestyle sessions lost more weight. Fasting glucose increased in all groups. FINDRISC score increased, but less so among high-risk men. (+)

Clinical significance estimate* the scoring is marked as follows: ++ significant reduction in DM risk; + significant improvement in (most) target risk factors; (+) significant improvement in some/few risk factors; (−) no effect

*Abbreviations: AHA* American heart association, *BL* baseline, *BMI* body mass index, *CG* control group, *CVD* cardio vascular disease, *DPP* Diabetes Prevention program, *E%* percentage energy from, *f-Glu* fasting plasma glucose, *FU* follow-up, *GDM* gestational diabetes mellitus, *GP* general practice, *HDL* high density lipoprotein, *HR* Hazard ratio, *IFG* Impaired fasting glucose, *IG* intervention group, *IGT* impaired glucose tolerance, *LDL* low density lipoprotein, *OGTT* oral glucose tolerance test, *PA* physical activity, *RCT* randomised controlled trial, *SMS* short message service, *T2D* Type 2 diabetes, *WHO* World Health Organization;

**Table 2:** Overview of the studies targeting participants age 18–45 years and with minimum follow-up time 6 months

Study	Reach		Implementation & adaptation		Efficacy & maintenance
Name/ acronym Country Reference	Target group, age range/mean	Screening and recruitment; attrition	Study design, lifestyle goals/targets	Intervention delivery	Results Clinical significance estimate*
Daqing Diabetes Prevention Study China Pan et al., 1997 [5] Li et al., 2008 [15]	People living in Daqing area, > 18 y old IGT	110,660 people screened with OGTT 577 randomized sex: 54% men age: 45 ± 9 y	Cluster randomized controlled study in four groups: control, diet only, physical activity (PA) only, or diet + PA FU 6 years (*n* = 533) + 20 years. Goals: Diet (increase vegetables, decrease alcohol and sugar, caloric and weight reduction if overweight), PA (1–2 units/day; e.g. 30 min of slow walking etc.)	Individual counselling + compliance evaluation by physician/nurse every 3 months + small groups weekly for 1 month, monthly for 3 months and every 3 months thereafter. Intervention duration was 6 years.	HRs (adjusted for baseline BMI and f-Glu): HR = 0.69 for diet vs CG, *p* < 0.03; HR = 0.54 for diet + PA, *p* < 0.0005; 0.58 (diet+PA), *p* < 0.005; HR = 0.41 for PA vs CG *p* < 0.0005. The average number of PA units per day was higher after 6 years. ++
Hoorn Prevention Study The Netherlands Lakerveld et al., 2012 [26] Lakerveld et al., 2013 [27]	Age 30–50 At least 10% risk for T2D and/or CVD estimated according formula of ARIC and SCORE projects.	A screening invitation to GP customers (*n* = 8193). 2401 respond, 921 eligible based on waist circumference. 772 were screened. *n* = 622 sex: 42% men age: 43.5 years	RCT in general practice. FU 12 months (*n* = 502). Goals were at least one fruit, at least 200 g vegetables and at least 30 min PA per day.	Participants were offered 6 face-to-face sessions (30 min) and 3 monthly telephone sessions with trained nurses. Methods were based on motivational interviewing, problem solving treatment, theory of planned behaviour and theory of self-regulation. CG got brochures of health guidelines.	No significant results in weight or fasting glucose or glucose tolerance. Increase in fruit intake between baseline and 6 months (1.1 - > 1.3 pieces per day) but not after 12 months. Median participation in sessions was 2. (−)
Zhiiwapenewin Akino’maagewin: Teaching to Prevent Diabetes, ZATPD Canada Ho et al., 2008 [57]	Native North Americans Native North Americans (=high T2D risk), non-pregnant, living in the community at least 30 days.	Screening from the community, IG *n* = 57, CG *n* = 38. *n* = 133 sex: 22% men age: 42 y	Non-random assignment of communities into intervention/ comparison FU 12 months (*n* = 95). Improve dietary choices (reduce fat and sugared drinks) and physical activity by increasing knowledge, self-efficacy, and attitudes.	Intervention was based on social cognitive theory of behaviour change and implemented in three components. School component with 16 + 17 sessions lead by teacher; children as “change agents”. Store component to support more appropriate foods. Community component, media involvement, cooking demos, community events, family fun nights in collaboration with existing health and social services.	Higher healthy food acquisition scores after intervention; no change in healthiness of food preparation scores. No change in BMI, decrease in PA in both groups (+)
Making the Connection Healthy Living Program, MTC HLP USA Ruggiero et al., 2011 [36]	underserved latino population; age 18–65 years; BMI > 24.9, normal glucose or prediabetes; Latin background	Community-based health screening events (schools, family centers, hospital etc). 1162 screened, 367 tested for eligibility, 244 eligible. *n* = 69, sex: 7% men age: 38 y	Single-group, non-randomized follow-up, community-based translation of DPP. FU 12 months (n = 57). DPP goals: Weight reduction 7%; Diet: fat 25 E% Physical activity (e.g. brisk walking) 150 min/week	Culturally specific intervention was developed and conducted in collaboration with the community to minimize barriers to participation education, literacy, language, income, transportation, lack of medical coverage. 22 group sessions during 1 year, delivered by trained community health workers. Cook books, pedometer, scales provided. Group walks arranged. Participants attended 57% of group sessions.	At 6 m, 20% achieved 7% weight reduction, and at 12 m 16% achieved. Moderate improvements in body weight (− 4.8 kg at 6 m, − 2.8 at 12 m), waist, fruit and vegetables, fat intake, PA were observed at 6 m but attenuated at 12 m. Forward movement in “stages of change” scale was observed. +
Families United USA Perez Siwik et al., 2012 [58] Kutob et al., 2014 [59]	Families with risk for T2D Diabetes risk factor (BMI > 25, inactivity, family history, etc.), no T2D, not pregnant, able to participate in group sessions	Community and clinic-based recruitment, 164 were interested, 108 screened *n* = 29 (+ 29 support people) sex: 26% men age: 45	Pretest-posttest study. Family-based intervention based on DPP. A household member/friend accompanied in the sessions. FU 12 months (*n* = 18) DPP goals (7% weight reduction, 150 min of PA/week); reduction in portion sizes and carbohydrates, especially sugared beverage, fat and fast food.	Patient-centered, multiculturally tailored intervention to elicit participants’ explanatory models regarding their diabetes risk. Physician+dietician delivered 12 group visits every 2 weeks over 6 months + 2 booster sessions. Cognitive behavioural approach aimed at increasing resilience (flexible thinking) skills. 15 min PA during each session. Attendance rate was 72% for the finishers.	Outcome measures were reduction in the total number of predefined diabetes risk factors (BMI, WC, BP, HbA1c, Insulin, GI, PA). Number of predefined risk factors reduced from 4.8 to 4.1 at 6 months and to 3.4 at 12 months, primarily due to reduction in GI and fasting insulin. +
“Diabetes prevention program in public housing communities” USA Whittemore et al., 2013 [60] Whittemore et al., 2014 [61]	People living in low-income public housing communities age > 21 y, 2 or more T2D risk factors (overweight, age, family history)	Convenience sample in 4 rural public housing communities. n = 67, sex: 79% female age: 40 y	Cluster-randomized implementation of DPP in low-income public housing communities. Enhanced standard care vs. mDPP, *n* = 67, diverse ethnicity (aim *n* = 100). FU 6 months (*n* = 48) DPP goals: Healthy eating plan, reduced calories, weight reduction 5–10%, physical activity 150 min/week	DPP program modified after focus groups. Two homecare nurses (8 h training) implemented the program and local community health workers (4 h training) assisted. IG got 7 interactive education classes during 6 months based on behavioural support on goal-setting, self-monitoring; problem-solving + gift-card raffles. CG got written information + two interactive education classes	No changes in body weight or other clinical risk factors, or behavioural or psychological outcomes. (−)
DH!AAN The Netherlands Admiraal et al., 2013 [48] Vlaar et al., 2012 [49]	South Asian migrants Age 18–60 y; IFG, IGT, HbA1c > 6,0% or HOMA-IR > 2.39	2307 screened via general practices (invitation letter with reply card), followed by reminder and telephone call). *n* = 536 age: 44.9 y sex: 49.4% men	RCT in general practice among South Asian migrants in Netherlands getting a culturally targeted intervention or generic lifestyle advice (control). FU 2 years (*n* = 335). Goals according to SLIM study; based on current guidelines on diet and physical activity.	6–8 individual sessions in general practice during 6 months, 2 sessions during the next 6 months + 1 family session + two cooking classes. 20-week supervised exercise program was offered. Trained dieticians gave dietary counselling using motivational interviewing. Participants got a gift coupon for participating in baseline measurements. Control arm got 2 group sessions + 2 flyers.	No significant results. Median participation in 5 individual sessions. High drop-out and low participation 26% participated in family session, 26% in cooking sessions and 22% in PA sessions. (−)
“Lifestyle modifications in Chinese women who had gestational diabetes mellitus” China Shek et al., 2014 [54]	Age > 18 years; GDM history; IGT 6–8 weeks after delivery; excluded if insulin for GDM	Patients from hospitals were invited if criteria were fulfilled *n* = 450; sex: 100% women age: 39 y	RCT conducted in hospital in Hong Kong. Intensive lifestyle intervention vs. no intervention (control). FU 36 months (*n* = 423). Optimal caloric intake (based on Harris-Benedict) for ideal body weight	7 individual sessions in 3 years (3 m, 6 m 12 m, 18 m, 24 m, 30 m, 36 m). Dietician and study nurse gave individual dietary and exercise advice based on food and exercise records (*n* = 7).	In women > 40 years the difference in cumulative incidence of diabetes between groups was significant. Significant differences at 1 y measurements in BMI and waist-hip ratios, but not significant at 3 y in the end of the study. (+)
Dulce Mothers USA Philis-Tsimikas et al., 2014 [62]	Low-SES Latinas Latina, 18-45y, GDM during past 3 years	263 contacted by information from medical records, 193 met criteria, 102 consented and came to lab *n* = 84 sex: 100% women age: 31.9 y	Single-group, pretest-posttest 6 m follow-up (*n* = 70) DPP goals: Weight reduction 7%, diet: fat 25 E%, physical activity (e.g. brisk walking) 150 min/week (700 kcal/week)	Condensed DPP based on social cognitive theory; trained peer educator lead educational group sessions, 8 sessions/8 weeks (core intervention) + additional monthly maintenance sessions e.g. weekly healthy lifestyle goals that involve the family members + discussions about culturally driven fatalistic health beliefs, mean attendance in 6 out of 8 sessions	No significant weight loss; however correlation between attendance and weight reduction. HbA1c increased slightly (5.73- > 5.82). Moderate improvement in cholesterol, LDL, triglyserides and diastolic BP. (+)

Clinical significance estimate* the scoring is marked as follows: ++ significant reduction in DM risk; + significant improvement in (most) target risk factors; (+) significant improvement in some/few risk factors; (−) no effect

*Abbreviations: BMI* body mass index, *BP* blood pressure, *CG* control group, *CVD* cardio vascular disease, *DPP* Diabetes Prevention program, *E%* percentage energy from, *f-Glu* fasting plasma glucose, *FU* follow-up, *GDM* gestational diabetes mellitus, *GI* glycemic index, *GP* general practice, *HR* Hazard ratio, *IFG* Impaired fasting glucose, *IG* intervention group, *IGT* impaired glucose tolerance, *mDPP* modified DPP, *OGTT* oral glucose tolerance test, *PA* physical activity, *RCT* randomised controlled trial, *WC* waist circumference

**Table 3:** Overview of the studies in the complementary search for studies published between January 2015 and January 2019

Study	Reach		Implementation & adaptation		Efficacy & maintenance
Acronym / Name of study Country References	Target group, Inclusion criteria	Screening, recruitment, study population (n, sex, mean age)	Study design, follow-up (FU) duration, lifestyle goals/targets	Intervention delivery, intervention duration, change theories	Results Clinical significance estimate*
Reaching Out and Preventing Increases in Diabetes (RAPID) USA Ackermann et al.,. 2014 [63] Ackermann et al., 2015 [64]	Economically disadvantaged adults ≥18 years old, BMI ≥ 24 kg/m2, no prior T2D, HbA1c level 5.7–6.9% or FPG > 100–125 mg/dl	12,787 patients were identified from 9 primary care clinic database; 3064 identified as high risk by primary care glucose tests; 640 attended screening visit. *n* = 509 (*n* = 252 for CG and *n* = 257 for IG) sex: 29.3% men age: 50.8 ± 12.2 y	Community-based randomized trial in economically disadvantaged adults. 2 groups: standard clinical advice plus a group-based adaption of the DPP offered by the YMCA, versus standard clinical advice alone. Follow-up: 12 months. Weight loss of 5–7%; moderate physical activity; lower dietary fat and total calorie consumption.	16 classroom-style behavioural counselling meetings, lasting 60 to 90 min and delivered over 16 to 20 weeks. Following monthly 60-min maintenance lessons until the end of the trial. YMCA offered limited guest-access and tools such as a step counter, measuring cups, fat and calorie tracking tools and recipe guides. Intervention was based on the DPP and included Goal-setting, self-monitoring and participant-centred problem solving.	Mean 12-month weight loss was 2.3 kg (95%CI: 1.1 to 3.4) more for the intervention arm than for standard care. Participants attending ≥9 lessons had a 5.3-kg (95% CI: 2.8 to 7.9) greater weight loss than did those with standard care alone. No significant differences in HbA1c, systolic blood pressure, HDL cholesterol or total cholesterol at 12 months. (+)
Diabetes mellitus and abnormal glucose tolerance development after gestational diabetes Spain Pérez-Ferre et al., 2015 [65]	Women with prior gestational diabetes Prior GDM, normal fasting glucose at 6–12 weeks postpartum	300 were invited *n* = 260 were included (130 in IG and 130 in CG) sex: 100% women age: 35 y (range 31–38 y)	RCT in a hospital setting, Mediterranean lifestyle intervention vs. control. Follow-up: 3 years (*n* = 237, 126 in IG and 111 in CG) For both groups: Mediterranean diet, physical activity and smoking cessation. Goals for IG: ≥5 servings (svgs) fruits and vegetables /day, > 2 svgs legumes/week, > 3 svgs nuts / week, daily use of virgin olive oil, ≥3 svgs oily fish / week, < 2 svgs red and processed meat / week and < 2 svgs non-skimmed dairy products / week.	Intervention group: 2-h group session at the 1st visit + 5 individual reinforcement sessions (45-min) at the hospital + supervised exercise program: group and individual sessions (1-h, 4 days per week) for 10 weeks 3–6 months post-delivery and 3 reinforcement sessions. Exercise: intensive supervised program. Control group: 2-h group session at the 1st visit and 3 annual follow-up visits.	Less women in the IG (42.8%) developed glucose disorders compared with the CG (56.7%), *p* < 0.05. Also significant reductions in BMI, waist circumference, insulin, HOMA-IR, LDL-cholesterol, triglycerides and Apo lipoprotein B in the IG compared with the CG. ++
Fit Body and Soul (FBAS) Study USA Dodani et al., 2009 [66] Williams et al., 2013 [67] Sattin et al., 2016 [68] Rhodes et al., 2018 [69]	African-American in Georgia area Age 20–64 years, self-described African American, BMI ≥25 kg/m^2^, no plans for moving, non-diabetic	Study- trained church health advisors distributed flyers to church members and made scripted-podium announcements to promote the study. 710 subjects from 20 churches located in a Georgia metropolitan area consented. *n* = 604 (*n* = 317 for IG and 287 for health education).	Single-blinded, cluster-randomized trial in African Americans. 2 groups: Fit Body and Soul intervention vs. health education (control). Follow-up: 12 months. Faith-based adaptation of the Group Lifestyle Balance program: weight reduction of ≥7% of initial weight and physical activity of ≥150 min per week of brisk walking.	Fit Body and Soul: The church health advisors held 12 weekly sessions comprised strategies to reduce calories and dietary fat, encourage physical activity, and behavioural modification such as stimulus control, goal setting, and problem solving followed by 6 monthly sessions. Health advisors phoned participants to review food and activity log and use scripted motivational interview messages to address participant barriers to lifestyle changes. Health education: 12 weekly sessions and then 6 monthly sessions, delivered by church health advisors including group discussion regarding health topics.	At 12 months, IG had a significant difference in adjusted weight loss compared with health education (2.39 kg vs. − 0.465 kg, *p* = 0.005) and were more likely to achieve a 7% weight loss (19% vs. 8%, *p* < 0.001). Fasting glucose did not differ between arms. In analyses with prediabetics only IG had a significant decline in fasting glucose compared to CG (− 12.38 mg/dl vs. + 4.44 mg/dl; *p* = 0.02). Per-person intervention cost was $442.22 for IG vs. $391.83 for CG per-person. +
Gestational Diabetes’ Effects on Moms (GEM) study USA Ferrara et al., 2014 [70] Ferrara et al., 2016 [71]	Women with GDM history Age ≥ 18 years, GDM diagnosis.	2480 identified; *n* = 2.280 (1087 in lifestyle intervention and 1.193 in usual care). sex: 100% women	Pragmatic cluster RCT of 2 groups in 44 medical facilities at Kaiser Permanente Northern California. Follow-up: 12 months (*n* = 1420). Reaching pregravid weight if pregravid BMI < 25 kg/m^2^ or losing 5% of pregravid weight if their pregravid BMI ≥ 25 kg/m^2^.	Intervention: 13 telephone sessions between 6 weeks and 6 months postpartum. Women were encouraged to set weekly goals for daily fat and caloric intake and to work up to 150 min of PA per week. Motivational interviewing and theoretical constructs from social cognitive theory and the transtheoretical model were used. 3 maintenance newsletters were mailed during 7–12 months post-partum. Usual care: 2 pages of lifestyle recommendations sent via mail.	IG had a 28% higher odds (95%CI: 1.10–1.47) of meeting postpartum weight goals than CG. Women who completed all 13 sessions had double odds (OR: 2.16, 95%CI: 1.52, 3.07). Fewer women in the IG developed prediabetes or diabetes than in CG. However, HR for did not reach statistical significance.+
Fair Haven Community Health Center’s Diabetes Prevention Program USA Van Name et al., 2016 [72]	Low-income Hispanic women Age 18–65 years, ≥ 1 risk factor for diabetes, OGTT.	1093 women identified as being at risk; 383 had prediabetes in OGTT. *n* = 130 age: 43 y sex: 100% women	RCT of 2 groups in low-income Hispanic women in Fair Haven community health centre Follow-up: 12 months (*n* = 122) Based on Diabetes Prevention Program: 7% weight loss (decreasing dietary fat and caloric intake) and ≥ 150 min per week of moderate-level physical activity.	IG: Family-centred 14-week group program with 1-h lifestyle class per week focusing on healthy food choices, behaviour change and weight loss led by a trained bilingual nurse. The curriculum was enhanced for a population with lower literacy with a hands-on learning approach including weekly cooking demonstrations, group learning sessions at the local grocery store, and encouragement to participate in the neighbourhood community farm. CG: 1 diabetes prevention counselling with nurse and dietitian.	The intensive intervention group lost 3.8 kg (4.4%), while the usual care group gained 1.4 kg (1.6%, *p* < 0.0001). 2-h glucose excursion decreased 15 mg/dL (0.85 mmol/L) in the intensive intervention group and 1 mg/dL (0.07 mmol/L) in the usual care group (*p* = 0.03). Significant decreases favoring intervention were also noted in BMI, percent body fat, waist circumference, and fasting insulin. +
Community-based HEalthy Lifestyle intervention Program (Co-HELP) Malaysia Ibrahim et al.,2016 [73]	18–65 years old, able to read and understand Malay or English, fasting blood glucose 5.6–6.9 mmol/L, and/or 2-h glucose 7.8–11.0 mmol/L in 75 g OGTT, BMI 23–39 kg/m2	Recruiting from the general population through healthcare providers and presentations at community-halls, mosques, and media. 685 were screened *n* = 268 (IG n = 122; CG *n* = 146) sex: 35.8% men age: 53 y	Quasi-experimental study with repeated measures, conducted in two sub-urban communities. 2 groups: intensive lifestyle intervention vs. standard care. Follow-up: 12 months (*n* = 236) Reduction of 5–10% of initial body weight for overweight and obese participants, reduction of calorie intake (20–25 kcal/kg body weight) and an increase from light to moderate physical activity (≥ 600 METs-minute/week).	IG received 12 90-min group sessions and ≥ 2 individual sessions with a dietitian and a researcher to reinforce behavioural change over 12 months. Sessions were first held more intensively (9 sessions /6 months) followed by 6 months maintenance phase with 3 monthly sessions (Sessions 10–12) and follow up through telephone calls or home visits for the last 3 months. Other group got standard care in primary health care.	IG mean fasting glucose reduced by − 0.40 mmol/l (*p* < 0.001), 2-h post glucose by − 0.58 mmol/l (p < 0.001), HbA1C by − 0.24% (p < 0.001) and waist circumference by − 2.44 cm (*p* < 0.05). Greater proportion of IG met the weight loss target (24.6% vs. 3.4%, p < 0.001) and physical activity of > 600 METS/min/wk. (60.7% vs. 32.2%, p < 0.001) compared to the CG. +
Lifestyle Modification in Information Technology (LIMIT) India Limaye et al., 2017 [74]	Employees in 2 IT industries ≥3 diabetes risk factors (family history of cardio-metabolic disease, overweight, high BP, IFG, high triglycerides, high LDL, low HDL).	437 employees in 2 multinational IT industries in Pune (India) were screened *n* = 265 (132 in CG and 133 in IG); age: 36.2 ± 9.3	RCT in 2 groups: Technology based lifestyle intervention Follow-up: 1 year (*n* = 203) 5% weight loss for overweight/obese; 4 lifestyle modification goals: exercise ≥150 min/week, intake of giber-rich foods ≥8 servings/week, intake of calorie-dense foods ≤4 servings/week and smoking cessation.	Before randomization, all participants attended a 1-h group session on lifestyle modification. Intervention group: information on lifestyle modification through 3 mobile phone messages and 2 e-mails per week for 1 year. Additional support was provided through a website and a Facebook page.	The prevalence of overweight/obesity reduced by 6.0% in the IG and increased by 6.8% in the CG (risk difference 11.2%; 95% CI: 1.2–21.1; *P* = 0.04). There were also significant improvements in lifestyle habits, waist circumference, and total and LDL cholesterol in the IG. +
Promotora Effectiveness Versus Metformin Trial (PREVENT-DM) USA Perez et al., 2015 [75] O’Brien et al., 2017 [76]	Socioeconomically disadvantaged Hispanic females in Philadelphia Hispanic, female, age ≥ 20 y, Spanish-speaking, BMI ≥23 kg/m2, prediabetes*	573 women contacted in community health fairs and at community health centers; 441 were at high risk (ADA score ≥ 4); 197 were screened; *n* = 92 (33 lifestyle, 29 metformin and 30 control); sex: 100% women age: 45.1 ± 12.5.	RCT in socioeconomically disadvantaged Hispanic women (Latinas). 3 groups: lifestyle intervention vs. metformin vs. control. Follow-up: 12 months (*n* = 65, lifestyle 30, metformin 27 and CG 28) Goals based on DPP: 5–7% weight loss by improving dietary patterns (decreasing fat and calorie consumption) and promoting moderate physical activity (≥150 min per week).	Group-based adaptation of the DPP intervention delivered by community health workers over 24 sessions (group size 5–9 participants, sessions lasting approx. 90 min). The first 14 sessions occurred weekly, and the final ten sessions took place biweekly and then monthly. Behavioural strategies such as goal setting, self-monitoring, stimulus control, and problem solving were used. Participants were provided with a digital scale, pedometer, measuring cups, and logs for tracking dietary intake and physical activity.	Post-hoc pairwise comparisons were significant for weight loss in lifestyle vs. standard care groups (−4.8 kg, *p* < 0.001) and lifestyle vs metformin (− 3.1 kg, *p* = 0.013), but not for metformin vs. standard care (− 1.7 kg, *p* = 0.3). Reduction in waist circumference was significantly greater in lifestyle than the standard care group (*p* = 0.001). Differences among groups in HbA1c did not reach statistical significance (*p* = 0.063). +
Diabetes Prevention Program - Group Lifestyle Balance (DPP-GLB) in community centers USA Kramer et al., 2018 [77]	Age ≥ 18 y, BMI ≥24 kg/m2, presence of prediabetes* and/or the metabolic syndrome	281 were screened *n* = 134 were enrolled; age: 62.5	Before-after study in 3 senior/community centers. Follow-up: 12 months and to 18 months (*n* = 118 at 12 months; *n* = 107 at 18 months) Goals Based on the DPP: 7% weight loss and increase physical activity to 150 min/week.	The Group Lifestyle Balance Program included 22 sessions delivered over a 1-year period (12 weekly sessions transitioning to monthly sessions) led by a lifestyle coach. Lifestyle coaches (2 registered dietitians and an exercise specialist) completed a standardized 2-day training workshop. A DVD of the initial 12 sessions, was developed to provide an additional option for program delivery.	At 12 months, a significant decrease in mean weight loss of, along with improvements in HbA1c, insulin, blood pressure and physical activity level. At 18 months significant improvements in mean weight waist circumference and physical activity. +
Prevention of Diabetes in Euskadi (Pre-DE) Spain Sanchez et al., 2012 [78] Sanchez et al., 2016 [79] Sanchez et al., 2018 [80]	Age 40–75 years; FINDRISC ≥14; high risk according OGTT.	14 primary health centres selected; 66,293 individuals identified; 4170 screened; 2128 at risk for diabetes; 1314 had OGTT *n* = 1088 enrolled (634 in CG, 454 in IG).	Cluster randomized clinical trial. Clusterded by primary health care centres to 2 groups. Follow-up: 1 + 2 years (*n* = 872 in 12-month and 956 in 24-months). Goals Based on the Diabetes in Europe-Prevention using Lifestyle, Physical Activity and Nutritional (DE-PLAN).	IG: Phase 1 consisted of intensive intervention through 4 1.5-h monthly educational sessions in small groups (10–15 patients) to encourage the adoption of healthy habits; Maintenance phase of regular contact with participants (at least once every 6 weeks) mainly via telephone calls from nurses. Control centers provided usual care.	Incidence of diabetes was 12.1% in the CG and 8.4% in IG, with an absolute difference of 3.8% (95% CI: 0.18 -7.4%, *p* = 0.045) and a relative risk reduction of 32% (0.68; 95% CI: 0.47–0.99, *p* = 0.048). ++
Kerala Diabetes Prevention Program (K-DPP) India Sathish et al., 2013 [81] Mathews et al., 2018 [82] Aziz et al., 2018 [83] Thankappan et al., 2018 [84]	Age 30–60 years; Indian Diabetes Risk Score value of ≥60; absence of diabetes in OGTT.	3689 individuals were contacted through home visits; 1529 had Risk Score value of ≥60; 1209 attended community-based clinics; *n* = 1007 were enrolled (500 in IG and 507 in CG). age: 46 y	RCT in community settings in 2 groups. Follow-up: 12 + 24 months (*n* = 964 at 24 months) Goals: Increasing physical activity; promoting healthy eating habits; maintaining appropriate body weight by balancing calorie intake and physical activity; tobacco cessation; reducing alcohol consumption; ensuring adequate sleep.	Adapted from the Finnish Good Ageing in Lahti Region (GOAL) program and the Australian Greater Green Triangle (GGT) Diabetes Prevention Project. IG got 15 group sessions over 12 months (1 session delivered by the research team, 2 sessions by local experts and 12 sessions by trained lay peer leaders), a handbook of peer support and its role in lifestyle modification and a workbook to guide self-monitoring of lifestyle behaviours, goal setting and goal review. Both groups got health education booklet.	At 24 months, diabetes developed in 17.1% in CG and 14.9% in IG (RR: 0.88, 95% CI 0.66–1.16, *p* = 0.36). IG had significantly greater reduction in Indian Diabetes Risk Score and alcohol use and a greater increase in fruit and vegetable intake and physical functioning score of the HRQoL scale. ++
Jew and Bedouin women with recent GDM in the Negev area. Israel Zilberman-Kravits et al., 2018 [85]	Jewish and Bedouin women with prior GDM prior GDM	307 women identified; *n* = 180 (103 in IG, 77 in CG). sex: 100% women age: 35,6 y *n* = 176 at 12-month follow-up; *n* = 104 at 24-month follow-up.	RCT in2 groups. Follow-up: 1 and to 2 years after baseline (n = 176 at 12-months; n = 104 at 24-months) Culturally adapted dietary and exercise recommendations for increase PA and decrease unhealthy foods.	The IG participated in healthy lifestyle sessions led by a dietician and a sports instructor for 24 months after delivery. Participants had 3 individual 45-min counselling sessions and 4 90-min group meetings (10 women each). Participants were given both verbal and written information, had the opportunity to practice physical activities during the meetings and received healthy meals that included low-fat products, such as yogurt, vegetables, fruits and whole-grains.	The intervention significantly reduced insulin, glucose and HOMA-IR levels compared with control (p < 0.001). Also significant differences in lipidemic profile, blood pressure and physical activity level between groups. +

*BL* baseline, *BMI* body mass index, *CG* control group, *CVD* cardio vascular disease, *DPP* Diabetes Prevention program, *E%* percentage energy from, f-Glu fasting plasma glucose, *FU* follow-up, *GDM* gestational diabetes mellitus, *GP* general practice, *HDL* high density lipoprotein, *HR* Hazard ratio, *IFG* Impaired fasting glucose, *IG* intervention group, *IGT* impaired glucose tolerance, *LDL* low density lipoprotein, *OGTT* oral glucose tolerance test, *PA* physical activity, *RCT* randomised controlled trial, *SMS* short message service, *T2D* Type 2 diabetes

*Prediabetes: fasting glucose 100–125 mg/dl and/or HbA1c 5.7–6.4%

Clinical significance estimate* the scoring is marked as follows: ++ significant reduction in DM risk; + significant improvement in (most) target risk factors; (+) significant improvement in some/few risk factors; (−) no effect

## Results

In the primary literature review of publications dated Jan 2000-Jan 2015, searches identified 663 potentially relevant publications, of which 80 studies met the initial inclusion criteria. For the first approach, altogether 27 studies targeted at population aged ≥18 years were reviewed after discarding the studies with the follow-up time under 12 months (Table 1). Of these, 12 were completed in Europe [6, 26, 29, 30, 32, 33, 35, 37, 41, 48, 55, 86], five in the USA [36, 44, 45, 50, 87], three in China [5, 47, 54], four in Japan [22, 28, 51], two in India [52, 88] and one in Australia [31].

In the second approach, the inclusion criteria of participants’ mean age ≤ 45 and follow-up at least 6 months were fulfilled only in nine studies, of which two were completed in Europe [26, 48], five in the USA or Canada [36, 57, 58, 60, 62], and two in China [5, 54] (Table 2). There were five studies which fulfilled inclusion criteria for both reviews [5, 26, 36, 48, 54].

The complementary search found 12 studies published after January 2015 (Table 3). In brief, six studies were conducted in USA [63, 64, 66–72, 75–77] (most were based on the DPP intervention implemented with adaptations in various settings), two in Spain [65, 78–80], two in India [74, 81–84], one in Israel [85] and one in Malaysia [73]. All 12 studies had a mean follow-up of at least 12 months and 5 included younger individuals (mean age ≤ 45 years old) [65, 72, 74–76, 85] and another three reported a mean participant age of ≤50 years old [66–71]. The core elements of the implementation of the high-risk intervention were identified through a synthesis of selected studies using RE-AIM model [14].

### The core elements of implementation

#### Reach

The total number of participants was over 13,000 in the studies with the participants aged ≥18 years and minimum follow-up of 12 months. Most common inclusion criteria in these studies were IGT or IFG based on laboratory tests (mentioned in 16 studies). In addition to laboratory tests, the FINDRISC diabetes risk score was used as an inclusion method in five studies [30, 32, 35, 37, 41] and used as a pre-screening instrument in one study [30]. Three studies used other risk scores (e.g. AUSDRISC) or risk algorithms for inclusion [26, 33, 44]. In addition, previous gestational diabetes was the basis of inclusion in one study [54], and BMI in another study [36]. It appears that none of the risk identification methods was superior as regards to the subsequent effectiveness of the intervention. The process of screening and recruitment was often reported to be laborious and more time-consuming than expected. When initial contact was not targeted at a risk population, the final inclusion was in general less than 10%, for example 0.5% in Da Qing [5], 2% in the Diabetes Prevention Program (DPP) [87] and approximately 10% in the Diabetes Prevention Study (DPS) [6]. The proportion of included subjects was lower in studies that had higher baseline risk (e.g. IGT) as an inclusion criterion. Only two studies stated that they were targeted at “underserved” or socioeconomically “vulnerable” population groups [36, 44]. Both of them showed significant improvement in target risk factors. In addition, two studies [37, 48] either included or were targeted solely at minority groups such as immigrants.

From the studies targeted at less than 45 years old participants, the major difficulties were related to participation. Lack of interest to take part in the studies was common and the drop-out rates tended to be in general high and participation in interventions in general inadequate. For example in The Families united study only 18 participants, of 90 eligible screened, attended the 12 month follow-up [58] and in DH!AAN only 22–26% of participants attended the lifestyle sessions. Some studies had completed focus group interviews and engaged the community already in the planning phase [49], but that did not have an unambiguous effect on the actual participation or the achieved lifestyle changes. Inviting people from e.g. registers as opposed to asking volunteers with self-perceived risk to sign up has been especially challenging and the response rate has generally been low. The participant selection method has also been reflected in attendance in interventions and evaluation measurements (especially in control groups or control areas), resulting in higher attrition. Five out of the nine studies, with the participants aged ≤45 years and the minimum follow-up of 6 months, were targeted at “vulnerable” population groups, such as native North Americans [57], public housing communities [60] or underserved Latino population in USA [36], or immigrants in the Netherlands [48]. The results from these studies were comparable to other 4 studies targeting general high diabetes risk population.

In complementary search 5 studies were targeted on vulnerable groups, one on economically disadvantaged adults [64], one on African-Americans in Georgia US [66], one on low-income Hispanic women [75], one on Socioeconomically disadvantaged Hispanic females in Philadelphia [75, 76] and one in developing country [81].

#### Adoption and implementation

In most of the studies with follow-up time over 12 months, the lifestyle goals were based on DPP or DPS and were related to body weight (reduction 5–7% recommended), changes in diet and increase in physical activity. Frequent dietary goals were to increase fibre, whole grains, fruit and vegetables, and to reduce total, saturated and/or trans-fat, sugar, refined carbohydrates, starch, alcohol and/or total calorie consumption. Also diet related lifestyle targets such as “reducing the frequency of eating out” were mentioned as target [22]. The studies differed from each other in relation to what and how much each target was emphasized. The participants in Chinese studies were not in general overweight at baseline, so weight reduction target was typical only for the subgroup of overweight or obese people [5]. In the PREDIMED-Reus study completed in Spain and emphasizing Mediterranean diet enriched with extra-virgin olive oil or nuts, there was neither weight reduction (despite high rate of obesity) nor physical activity target but yet a significant reduction in diabetes risk was observed [33].

Coach-delivered, face-to-face, individual (*n* = 11), group (*n* = 7), or group-individual-combination (*n* = 5) interventions were the most common delivery modes. In addition, in one study [28], intervention was delivered in hospital (in-patient) and in one study [37] intervention personnel went to the family homes. Short message system (SMS) was used in two studies [47, 52] as the primary intervention method. In addition, some studies used phone calls and telefax. SMSs proved to be a promising way to deliver intervention in a cost-effective way. The intensity of intervention (number and frequency of counselling sessions/contacts between the personnel and the participants) appeared to be more important for effective intervention than the mode. The coach has most often been either a nurse or a dietician. As long as the coach is trained appropriately to do the intervention and applies a structured intervention curriculum, there does not seem to be a big difference between professions.

Several theories of behaviour change were applied as the basis for the interventions. The most often mentioned theory was the stages of change/trans-theoretical model [24, 36, 41, 51, 52, 89] (applied in 6 interventions), followed by the theory of self-regulation (in three interventions) [26, 30, 31], the theory of planned behaviour (in 2 interventions) [26, 47], social cognitive theory of behaviour change (in two interventions) [44, 47], and the health action process approach, HAPA (in one intervention) [31]. None of the theories seemed to be unequivocally better or worse than the other and many of the highly successful studies have not stated specifically relying on an overarching theory of behaviour change. The reason for this might be that even though the theories have differing background presumptions about the process of behavioural change they utilize more or less the same components, techniques and tools, such as motivational interviewing, social and peer support, interactive learning strategies, behavioural support on goal-setting and self-monitoring, problem-solving and feedback. Of the two studies using SMS, the Indian study [53] utilized the tailoring of the messages based on individuals’ estimated stage of change and showed significantly better results compared with the Chinese study [47] using generic messages. The studies where motivation of participants was emphasized were successful. In many studies, self-monitoring and personal goal setting were seen as very important. In less successful studies there was a high drop-out rate, which might be related to motivation. In the studies targeted at less than 45 years old participants, the results of the intervention did not seem to depend on the theoretical framework or, whether there was a theoretical framework mentioned at all. However, several authors still emphasized the need for a theoretical framework and structured intervention. Most studies, however, described components, techniques, and tools that are included in many of these theories.

The feedback from those who actually participated in the interventions was in general very positive. The authors’ recommendation in several studies was not to cut down the number of contacts/sessions and topics, as this would lead to dissatisfaction by the participants, but to increase the number of sessions and offer the sessions with shorter intervals, paying special attention to accessibility such as timing of the sessions and e.g. offering child care when needed. Also offering a variety of intervention modes (individual, group, SMS, telephone, DVD, internet) to choose from was considered a feasible strategy to reach participants in studies with mean age under 45 years old. Incentives for participation were recommended by some authors. Community partnership was considered important to train and support community health workers. However, employing full-time project staff as opposed to expecting local community workers to do the project in addition to their normal work was emphasized. One study (Families United) [58] aimed at recruiting a family member as a support person but that proved to be a challenge.

The complementary search showed that the basic contents of the interventions in the recent studies had not changed from the first diabetes prevention projects. Most studies applied traditional intervention modes, i.e. group and/or individual counselling sessions based on behavioural change techniques delivered by health professionals or trained non-medical community members. As reported in previously published studies, non-medical individuals delivered efficient interventions. Many studies highlighted the importance of attendance for intervention efficacy and the need of strategies to increase and sustain patient engagement; among studies targeted on women with GDM and on people with low socioeconomic status, adequate attendance was promoted through family involvement and childcare offer [70–72]. Also the community, in many innovative ways, was utilised as an intervention or recruiting place, for example the church as an intervention state for African-Americans [66], a workplace for employees in information technology industry as the recruiting and intervention venue [74] and peer-led intervention as a way to involve community in a study in India [84]. In the study using telephone/newsletter [70, 71] and the study using SMS/email technology [74] to deliver the intervention the results were comparable with others. Lifestyle advice through telecommunication was considered as an efficient, low-cost and potentially scalable intervention for technology-literate individuals. Even though different mobile phone applications, activity trackers and other modern technology have become widely available, the technology was not systematically used in modern studies.

#### Efficacy and maintenance

Of the 27 studies including the participants aged ≥18 years old and minimum follow-up of 12 months, eight [5–7, 22, 23, 28, 33, 40] were rated highly successful and showing meaningful reduction in diabetes incidence. In seven studies [30, 32, 35, 36, 44, 45, 50], meaningful improvement in (most) target risk factors were seen; in those studies reduction of diabetes incidence was either not a target or not achieved. In eight studies [29, 37, 41, 51, 54, 55], meaningful reduction in some/few risk factors was achieved. Only four studies [26, 31, 47, 48] failed to show any effect on risk factors or diabetes risk. In most studies including younger participants (18–45 years old), achieved results/changes in predefined outcome variables were less-pronounced than in studies with older participants. The exception for this rule was the Chinese Da Qing study [5], where a significant and highly meaningful reduction in diabetes risk in 6 years was achieved. However, also in that study there was no reduction in body weight in general, and the measured changes in diet and physical activity were modest. In general the achieved lifestyle changes were minor compared with changes in the studies on older participants. This might be due to lower self-perceived risk and life situation in general, such as “demands of motherhood and family life” as stated in one of the studies [54].

In the complementary search significant reduction in diabetes risk was distinguished in the two studies conducted in Spain of all 12 reviewed studies [4, 79, 80]. Both of these studies were conducted at a medical environment (primary health-care centres or hospital) by medical personnel, applied intensive interventions in terms of patient contact and reinforcement and had a long follow-up period of 2 and 3 years. In 8 studies significant reductions in most of targeted diabetes risk factors were achieved but reduction in diabetes risk was not stated. In two studies significant reductions were achieved only in some diabetes risk factors [63, 64, 75, 76]. In those studies, diabetes risk assessment for recruitment was based on Hb1Ac levels, which may have led to recruiting participants with relatively low baseline fasting glucose values and without room for improvement. Significant changes in glycaemic control were not seen, although interventions were efficient in achieving weight loss and improving body composition.

## Discussion

The systematic literature reviews revealed and highlighted several important aspects that were subsequently taken into account while developing the Feel4Diabetes high-risk intervention. To improve effectiveness as well as sustainable adoption and implementation of interventions, they should be targeted at people with increased type 2 diabetes risk. Risk identification can be based on fasting or 2 h blood glucose measurement, however, also non-invasive methods can be used. Since the publication of the FINDRISC in 2003 it has been used in several studies as the first-line or even sole risk screening tool. In the major type 2 diabetes prevention trials [6, 90] the oral glucose tolerance test was used as the screening method and IFG or IGT as inclusion criteria. In the studies included in this review, the risk identification method appeared not to be associated with the effectiveness of the intervention. The selection of risk identification method may thus be based on pragmatic issues such as cost, acceptability, and accessibility, especially when completing an implementation project. Of note, the measurement of glycated haemoglobin (HbA1c) has been found clearly inferior to oral glucose tolerance test (OGTT) in identification of prediabetes based on the meta-analysis by Barry et al. [91]. An important finding was that the process of screening and recruitment is often laborious and more time-consuming than expected; especially people with lower socioeconomic status may require additional effort and action. Thus, screening is a critical step of preventive interventions and should not be overlooked. Most studies included in this review show that changes in diabetes risk factors are similar regardless of whether the intervention is delivered by experts (clinically trained health professionals) or lay educators; therefore, costs associated with diabetes prevention can be lowered without sacrificing intervention effectiveness, involving nonmedical personnel.

Equally important is to arrange the intervention so that it is easily and conveniently accessible. In the younger age-group, the most important reasons for non-participation were lack of time and difficulties in participating in the scheduled counselling sessions. Furthermore, in most studies including younger participants (18–45 years old), achieved results/changes in predefined outcome variables were less-pronounced than in studies with older participants. Obvious reason for this is that a longer follow-up is needed to see effect on diabetes risk in younger people. The complementary literature search showed that the same challenges of recruitment and participant engagement continue in recent studies as in previous studies. Wider use of modern technology could help participants to commit on the intervention and the use of different community settings were seen helping the recruitment. Even in the most recent studies technology was quite rarely used, even though it was seen as efficient as the classic face-to-face counselling. Targeting intervention earlier in life might be a trend in most recent studies, 40% of studies conducted between 2015 and 2019 was targeted to people under 45 years old. Technology could be part of a solution to engage and reach younger participants.

The focus and general goal of intervention should be clearly specified and communicated. Cultural adjustments to the intervention goals probably increase the participation and motivation to make the suggested lifestyle changes. The intensity of intervention (number and frequency of counselling sessions/contacts between the personnel and the participants) is more important for effective intervention than the mode of delivery. Moderate but comprehensive changes in several lifestyles seem to lead to a good intervention effect. At least 3 years follow-up seemed to be required to show actual reduction in diabetes risk in high-risk individuals.

Theoretical model is considered important as a framework for the intervention. However, as long as it facilitates the understanding of the complexity of behavioural change, it doesn’t matter which model is used. Tools and methods shown to be efficacious include motivational interviewing, social and peer support, interactive learning and motivation and self-efficacy building strategies, support on individualized goal-setting, self-monitoring, problem solving, relapse management, and feedback.

Importantly, research on prevention interventions targeted at younger adults or vulnerable population groups such as people with lower socioeconomic position proved to be surprisingly scarce. More research is warranted, and Feel4Diabetes is an important example of projects aiming to fill this research gap.

## Conclusions

This narrative review highlighted several important aspects that subsequently guided the development of the Feel4Diabetes high-risk intervention. Research on diabetes prevention interventions targeted at younger adults or vulnerable population groups is still relatively scarce. Feel4Diabetes is a good example of a project aiming to fill this research gap.

## Data Availability

Not applicable.

## Abbreviations

BMI: Body mass index
DPP: Diabetes Prevention Program
DPS: Diabetes Prevention Study
HAPA: Health Action Process Approach
HbA1c: Glycated haemoglobin
IFG: Impaired fasting glucose
IGT: Impaired glucose tolerance
OGTT: Oral glucose tolerance test
RE-AIM: Reach, Effectiveness, Adoption, Implementation, and Maintenance
SMART goals: Specific, Measurable, Attainable, Realistic and Timely goals
SMS: Short message system
